# The Prevalence and Psychosocial Correlates of Ketum (*Mitragyna speciosa*) Use among Individuals on Methadone Maintenance Therapy Programme in Hospital Taiping, Malaysia

**DOI:** 10.3390/healthcare10040746

**Published:** 2022-04-17

**Authors:** Ling Ling Choo, Muhammad Muhsin Ahmad Zahari, Seng Kit Choy, Naemah Abdul Rahim, Rusdi Abd Rashid

**Affiliations:** 1 Department of Psychological Medicine, University Malaya Medical Centre, Kuala Lumpur 59100, Malaysia; lingling_choo@hotmail.com (L.L.C.); muhsin@ummc.edu.my (M.M.A.Z.); 2 Department of Psychiatry and Mental Health, Hospital Taiping, Taiping 34000, Malaysia; choysk1228@hotmail.com (S.K.C.); khairunnaem@yahoo.com (N.A.R.)

**Keywords:** ketum, kratom, methadone maintenance therapy

## Abstract

Ketum use is significantly prevalent amongst individuals in the northern states of Peninsular Malaysia. This study aims to investigate the prevalence and psychosocial correlates of Ketum use in individuals who are in the Methadone Maintenance Therapy (MMT) Programme at the Hospital Taiping. This is a cross-sectional study conducted in the methadone clinic at the Hospital Taiping. The study instruments used were Subjective Opiate Withdrawal Scale (SOWS), Alcohol, Smoking and Substance Involvement Screening Test (ASSIST) questionnaire, and Kratom Dependence Scale (KDS). A total of 215 subjects were recruited for this study. The prevalence of ketum users was 49.3% (*n* = 106). Chinese and Indian ethnicity had a lower tendency to use ketum compared to Malay ethnicity, with OR = 0.386 (95% CI 0.134, 1.113) and 0.119 (95% CI 0.035, 0.408), respectively. Individuals who used other illicit drugs had a higher tendency to use ketum with the adjusted OR = 9.914 (95% CI: 1.109, 88.602). Every one unit increase in SOWS increased the odds of being a ketum user by 1.340 (95% CI: 1.070, 1.677), whereas every one unit increase in duration in the MMT programme reduced the odds of being a ketum user by 0.990 (95% CI: 0.982, 0.998). Ketum use is prevalent amongst those in the MMT programme in this study. The high prevalence of ketum use is of concern and further interventions should be carried out to address this.

## 1. Introduction

Ketum leaf or kratom or *Mitragyna speciosa* (MS) is a native tropical plant that grows in Southeast Asia and has been used as a traditional medication by the locals [1,2,3]. It can be chewed, taken with drinks, or smoked. [4]. However, there is also a trend of ketum being used as a substitute recreational drug amongst youngsters [5]. There was a study performed in the northern states of Malaysia that demonstrated 88% reported daily use of ketum [6].

Ketum contains a cocktail of naturally occurring psychoactive alkaloids, such as mitragynine, 7-hydroxymitragynine, speciociliatine, and corynantheidine, which are known to be pharmacologically active [7]. Some data suggest that some of these alkaloids target opioid receptors, and behave as mixed opioid receptor agonists/antagonists [8]. However, these indole alkaloids are structurally and pharmacologically distinct from other opioids, hence producing partially overlapping but different effects [8]. Additionally, mitragynine appears to have an additional analgesic effect via the activation of alpha-2 adrenergic receptors, inhibition of COX-2, and calcium channel blockade [9,10]. Mitragynine also has some affinity to other receptors, such as serotonin, dopamine, and adenosine receptors. However, the physiological significance is unclear [8]. Physiologically, ketum has shown to have some anti-nociceptive, anti-inflammatory, and gastrointestinal (suppression of food and water intake) effects [11].

Overall, a small dose of ketum is similar to the effect of a stimulant, whereas at higher doses it has ‘opioid-like’ effects [12]. There have been local studies looking into ketum users in Malaysia and their reasons for use [6,13,14,15]. Reasons for usage of ketum include stamina and endurance, recreational use, improving sexual performance, helping with sleep, medicinal purpose, and reducing withdrawal symptoms [6]. Studies specifically involving current or former drug users who also use ketum show how these groups of individuals who used ketum are able to manage withdrawal symptoms and to abstain from using other opioids [13,15].

The MMT programme was established in Malaysia by the Ministry of Health Malaysia in October 2005 as part of a harm reduction programme aimed at opioid (especially heroin) abusers. The programme aimed to reduce the risk of bloodborne infections (e.g., HIV, Hepatitis B, and Hepatitis C infections) caused by the sharing of needles [16]. Studies have shown that the MMT programme has been successful in improving quality of life, protecting seronegative patients from getting new bloodborne infections, and improving social functioning and psychological symptoms [17,18]. However, it is also shown that there is a significant proportion of patients on the MMT programme who also use other psychoactive substances besides heroin, such as methamphetamine, cannabis, and benzodiazepines [19].

Ketum is widely used in Northern areas of Malaysia. Up to date, there have been no studies that evaluate the prevalence and psychosocial correlates of ketum use in individuals on the MMT programme. The authors chose to conduct the study on individuals on the MMT programme as ketum might affect the effectiveness of methadone therapy for those who are concurrently using ketum. Therefore, this study aims to determine the prevalence of ketum use in the MMT programme in Hospital Taiping. Another aim is to examine the psychosocial correlates of ketum use in individuals on the MMT programme in Hospital Taiping. This study also aims to identify the association between methadone dose and the severity of dependence on ketum use in the MMT programme. With this knowledge in hand, the authors would be able to tailor additional services to support these individuals.

## 2. Materials and Methods

A cross-sectional study was conducted amongst the individuals in the MMT programme Hospital Taiping from mid-August to November 2021. This study was approved by the Medical Research and Ethics Committee (MREC) with the NMRR ID of NMRR-21-1358-59203. A convenient sampling method was used in this study. Individuals who fulfilled the inclusion and exclusion criteria, and after informed consent signed were recruited for the study. The inclusion criteria were (i) all methadone users who are registered at the Hospital Taiping methadone clinic who have been on MMT for at least two months duration; and (ii) have the capacity to give written consent. The exclusion criteria were (i) under the age of 18 years old; (ii) currently hospitalized or have suicidal intent. The authors set a minimum of two months duration on the MMT programme to give adequate time for the methadone users to be ready emotionally and psychologically during the induction phase of the methadone programme. The authors excluded those who are currently hospitalized or have suicidal intent as they are either physically or psychologically not well which might affect the capability to answer the questionnaires provided in this study. Based on sample size calculation [20], a total of 215 subjects were recruited for this study, with a margin error of 5% and a confidence interval of 95%.

### 2.1. Measurement Tools

There were three sets of measurement tools used in this study. The SOWS and ASSIST questionnaires were administered to all the study subjects. The KDS was performed on only those with ketum use.

#### 2.1.1. Subjective Opiate Withdrawal Scale

The Subjective Opiate Withdrawal Scale (SOWS) is a valid and reliable instrument used to measure the severity of opiate withdrawal symptoms. It is a self-administered scale that contains 16 items. Each item is rated on a five-point Likert scale, from 0 (not at all) to 4 (extremely), corresponding to how the subject feels about each symptom right now. Scale 0 = not at all; 1 = a little; 2 = moderately; 3 = quite a bit; and 4 = extremely. The total score is the sum of the 16 items, ranging from 0 to 64. The higher the score indicates greater the severity. [21]. Besides that, the validated Malay version of SOWS is also available [22].

#### 2.1.2. Alcohol, Smoking and Substance Involvement Screening Test Questionnaire

The Alcohol, Smoking, and Substance Involvement Screening Test (ASSIST) was developed by the World Health Organization (WHO). It is a questionnaire used to screen for all levels of risky substance use in individuals [23]. The ASSIST questionnaire is an interviewer rated tool. The ASSIST consists of eight questions covering ten groups of substances, including tobacco, alcohol, cannabis, cocaine, amphetamine-type stimulants, inhalants, sedatives or sleeping pills, hallucinogens, opioids, and other drugs. The specific substance score will be recorded and will be grouped into low (0–3), moderate (4–26) and high risk (27+) levels. The risk score determines the level of intervention for the respondent, namely low risk (no intervention); moderate risk (receive brief intervention); and high risk (more intensive treatment, namely brief intervention with referral) [24]. The Malay version of the ASSIST questionnaire used was validated in a study among alcohol users [25].

#### 2.1.3. Kratom Dependence Scale

The Kratom Dependence Scale (KDS) was developed by Darika Saingam from Prince of Songkhla University, Thailand in 2014. It is a self-administered rating scale particularly designed to assess the degree of severity of ketum dependence among ketum users. The KDS contains 16 items, including preoccupation with use (items 1 to 4), impaired control overuse (items 5 and 6), continued use despite harmful effect (item 7), persistent desire or compulsion to use (items 8 to 11), withdrawal (items 12 to 14), and tolerance (items 15 and 16). It is a four-point Likert rating scale, from 0 (never) to 3 (always). The total score of KDS is from 0 to the maximum of 48, which is categorized into 3 score groups corresponding to the dependence severity. The total score of 0–13 (low score group) indicates low dependence; a score of 14–34 (medium score group) indicates moderate dependence; and a score of 35–48 (high score group) indicates high dependence [26]. To date, the KDS is the only validated rating scale for evaluating ketum dependence severity among ketum users. The Malay version of KDS for use in assessing dependency among kratom users was validated in 2018 [27].

### 2.2. Statistical Analyses

The Statistical Package for the Social Sciences (SPSS) version 26 software (IBM, Armonk, NY, USA) was used to analyse the study data. Descriptive analysis was done using mean and standard deviation for normally distributed data (Z values of skewness and kurtosis ≤ 3.29), whereas median and interquartile range were used for non-normally distributed data (Z values of skewness and kurtosis > 3.29) [28]. For categorical independent variables, frequency and percentage were presented. Spearman rank correlation was used to evaluate the association between SOWS, methadone dose and KDS. Chi-square test was used to compare two or more variables of categorical data, whereas the comparison of numerical variables was done using either independent t-test or Mann–Whitney U test depending on the normality of the data. All the factors significantly associated with ketum use in the individuals in the MMT programme were further tested using multiple logistic regression. A multicollinearity test was carried out to identify the potentially high collinearity issue prior to multivariate analysis. The alpha level of 0.05 was used. All data analyses were two-tailed.

## 3. Results

A total of 215 subjects were recruited from those undergoing the MMT Programme in Hospital Taiping. All 215 samples were involved in the data analysis. It was shown that age and duration of heroin use before starting on MMT were normally distributed. On the other hand, monthly household income, duration in the MMT programme, and SOWS violated the normal distribution.

### 3.1. Socio-Demographic Data

Table 1 displays the sociodemographic data and the clinical related variables of the subjects. The average age of the subjects was 45 years old, with a standard deviation of 11.54. The majority of the subjects were male (97.7%). About two-thirds of the subjects were Malay and of the Islamic religion. A total of 67.4% of the subjects were either single, divorced, separated, or widowed. Approximately 76% of the subjects were employed, and 84.2% of the subjects were staying with their family or friends. The median monthly household income was RM 1500.00, with an interquartile range of RM 1000.00. It is reported that 41.4% of the subjects completed at least secondary education. The median duration of subjects being in the MMT programme was 58 months with an interquartile range of 90 months. The median current methadone dose was 65 mg with an interquartile range of 40 mg. The average duration of heroin use before starting on MMT programme was 18.5 years, with a standard deviation of 11.09. Around three-quarters of subjects were imprisoned in the past. More than 90% of them had no psychiatric illness. Less than 3% of subjects had either Hepatitis B or HIV, whereas almost half of them have Hepatitis C. More than 90% of subjects have used other illicit drugs. The majority of the subjects did not have a high-risk score in the ASSIST. The median of SOWS was 0, with an interquartile range of 1.

### 3.2. Prevalence of Ketum Use

Table 2 shows the comparison of sociodemographic data and clinical related variables between ketum users and non-ketum users. A total of 106 subjects were ketum users, which comprised of 49.3% of the total subjects. The average age of non-ketum users was 50.3 years old, whereas the average age of ketum users was 39.5 years old, with a standard deviation of 10.40, and 10.02 respectively. Both non-ketum and ketum users were predominantly male. Majority of ketum users comprised of Malay ethnicity (88.7%) and Islam religion (91.5%). More than two-thirds of both non-ketum and ketum users were either not married, separated, divorced, or widowed. A total of 72.5% of non-ketum users, and 80.2% of ketum users were working. Next, 78.9% of the non-ketum users and 89.6% of ketum users were staying with their family or friends. Both groups had the same median of the monthly household income of RM 1500.00. Most ketum users completed secondary and tertiary education (55.7%) as compared to the non-ketum users (27.5%). The median methadone dose in both groups was similar (60 mg in non-ketum group; 70 mg in ketum group). Non-ketum users were in the MMT programme for a longer duration (median duration of 94 months) as compared to ketum users (median duration of 36.5 months). The average duration of heroin use before starting on MMT in non-ketum users was 21.5 years, with a standard deviation of 11.14, as compared to ketum users with the average duration of 15.4 years with the standard deviation of 10.19. A similar proportion of both ketum and non-ketum users were imprisoned before (more than 70%). The majority of both ketum and non-ketum users reported no history of psychiatric illness (more than 90%). Almost all subjects in the ketum and non-ketum groups did not suffer from Hepatitis B and HIV. A higher number of subjects had Hepatitis C amongst the non-ketum users (57.8%) as compared to the ketum users (35.8%). A large majority of subjects in both the non-ketum and ketum groups reported using other illicit drugs (84.4% amongst non-ketum users, and 99.1% amongst ketum users). The majority of both non-ketum and ketum groups did not have a high-risk score in ASSIST. The median of SOWS for both non-ketum and ketum groups was 0, but the ketum group had a higher interquartile range compared to the non-ketum group.

### 3.3. Correlation between SOWS, Methadone Dose and KDS

In Table 3, the Spearman ranked correlation test showed that there was a positive correlation between SOWS and KDS (rho = 0.570, *p* < 0.01). There was also a negative mild correlation between methadone dose and KDS (rho = −0.106, *p* > 0.05), but this was not statistically significant.

### 3.4. Univariate Analysis of Factors Associated with Ketum Use

Univariate analysis of the factors associated with ketum use is illustrated in Table 4. For ethnicity, Malay was selected as the reference group to compare with Chinese and Indian, as a large majority of ketum users in this study were of Malay ethnicity (88.7%), whereas Chinese and Indian ethnicity consist of 7.5% and 3.8%, respectively. Age, ethnicity, religion, accommodation, education, duration in the MMT programme, current methadone dose, duration of heroin use before starting on MMT, hepatitis C, other illicit drugs, high-risk score in ASSIST, and SOWS were significantly associated with ketum use at 0.05 level. These variables (except for religion) were subjected to a multicollinearity test before being included in the downstream multivariate analysis. Since ethnicity and religion are highly related, religion was not chosen to be included in the multicollinearity test.

### 3.5. Multivariate Analysis of Factors Associated with Ketum Use

The multicollinearity testing showed that age had a variance of inflation exceeding five. This concluded that there was a substantial-high collinearity issue between the variable age and other independent variables which likely affect the model stability in the multivariate analysis. Hence, age was excluded in the multivariate analysis.

Table 5 shows the multivariate analysis using binary logistic regression model on factors associated with ketum use. Out of ten factors involved, ethnicity, duration in MMT programme, other illicit drugs use, and SOWS remained significantly associated with ketum use after being adjusted by the other variables included in the multivariate analysis. Chinese and Indian ethnicity had a lower tendency to use ketum compared to Malay ethnicity, with OR = 0.386 (95% CI 0.134, 1.113) and 0.119 (95% CI 0.035, 0.408), respectively. Besides that, every one unit increase in duration in the MMT program reduced the odds of being a ketum user by 0.990 (95% CI: 0.982, 0.998). Subjects using other illicit drugs were 9.9 times more likely to use ketum compared to those who did not use other illicit drugs (95% CI: 1.109, 88.602). Last but not least, every one unit increase in SOWS increased the odds of being a ketum user by 1.340 (95% CI: 1.070, 1.677). The Hosmer–Lemeshow test showed that the data could fit in the model adequately (Chi-square = 5.863; df = 8; *p* = 0.663). Overall, this multivariate model was able to explain a total of 45.5% (Nagelkerke R square = 0.455) of variance among participants included in this survey.

### 3.6. Descriptive Statistics of Other Illicit Drugs

Table 6 shows the descriptive statistics of other illicit drugs. Among the five categories of illicit drugs, the highest number of illicit drugs used were stimulants (61.5% of non-ketum users, 91.5% of ketum users), followed by cannabis (58.7% of non-ketum users; 82.1% of ketum users), then sedative/ hypnotic agents (38.5% of non-ketum users; 42.5% of ketum users), then inhalants (2.8% of non-ketum users, 21.7% of ketum users), and lastly hallucinogens (7.3% of non-ketum users and 17% of ketum users).

## 4. Discussion

This study demonstrated a high prevalence of ketum use (49.3%) among a total number of 215 subjects who are in the MMT programme in Hospital Taiping. Up to date, there are no specific studies that capture the prevalence of ketum use among participants of the MMT programme in Malaysia. There have been instead several studies documenting the prevalence of ketum use in other groups in the population. A study which was conducted among rural settlers in Perlis, northern Malaysia found that 35 (24.3%) out of a total of 144 study subjects reported the use of ketum in their lifetime [29]. Another study at a drug rehabilitation centre in Kelantan, Malaysia demonstrated that the prevalence of ketum use was 16.8% [30]. Meanwhile, a Southern Thailand study involving 414 drug users from three different provinces showed that 85.2% of subjects were active ketum users [31]. In the west, a cross-sectional study conducted in the United States involving a total of 59,714 respondents demonstrated an estimated prevalence of past-year ketum use in adults of 0.8%, in contrast a lifetime prevalence of 1.3% [32].

Malays were found to be more likely to use ketum compared to Chinese and Indians in this study. A large majority of ketum users in this study are of Malay ethnicity (88.7%), whereas Chinese and Indian ethnicity consist of 7.5% and 3.8%, respectively. This is similar to the results of other studies. A study conducted in northern states of Malaysia; showed that out of 530 respondents who used ketum, 522 (98.5%) were of Malay ethnicity as compared to five (0.9%) of Chinese and three (0.6%) of Indian ethnicity [6]. Another study that recruited 204 subjects from opioid users with ketum use from the northern state of Penang in Peninsular Malaysia showed that 191 (94%) were of Malay ethnicity as compared to 13 (6%) non-Malay [15]. This could be because there is a higher proportion of Malays amongst the population in northern states in Malaysia including Taiping [33].

This study also demonstrated that a longer duration an individual is in the MMT programme will reduce the odds of being a ketum user. Methadone is the most commonly used medication for treating opioid use disorder [34]. A Cochrane systemic review showed that methadone appeared to be significantly more effective than non-pharmacological methods in reducing opioid use (RR = 0.66; 95% CI: 0.56, 0.78) [35]. A retrospective study was carried out in Hospital Raja Perempuan Zainab II, Kota Bharu, Kelantan involving 117 registered MMT individuals, showed that there was a significant reduction in heroin use after 12 months of the MMT programme, with the mean difference of 2.01 (95% CI: 1.45, 2.56) in heroin Q score Opiate Treatment Index [36]. Other studies showed that the longer duration of the MMT programme had better intervention outcomes, with better results in reducing illicit substances use [37,38,39,40]. A two-year study done in a methadone clinic in Malaysia showed that there was a reduction in the number of positive urine tests for each type of illicit substance, which included opioids, THC, amphetamine and methamphetamine, benzodiazepine, and MDMA, with a total reduction of the number of illicit substances from 12.7% to 8.3% two years after the initiation of MMT programme [37]. Other studies have also demonstrated that overall use of cocaine, amphetamine, benzodiazepine, and cannabis declined with the long-term MMT [38]. This reflects the efficiency of MMT programmes which could not only reduce opioid use, but also indirectly reduce other drugs use.

In this study, subjects who used other illicit drugs were 9.9 times more likely to use ketum, compared to those who did not use other illicit substances in the MMT programme. Many studies demonstrated that ketum use is associated with other illicit substances use [13,15,31,41,42]. Ketum is commonly used in combination with other illicit drugs. A study by Talek et al. showed that more than half of a total of 414 subjects used ketum in combination with other illicit drugs (such as opioids, methamphetamine and cannabis) [31]. Talek et al. also showed that amongst the different types of illicit drugs used with ketum, methamphetamine was demonstrated to be the highest [31]. This finding is similar to the findings of this study. Another study by Likhitsathian et al., demonstrated that 22 (11.3%) out of all 195 respondents who have used ketum in the past 12 months, were concurrently using other illicit substances, including stimulants, cannabis, and inhalants [43]. It is also not uncommon for the concurrent use of other illicit drugs among individuals who are in the MMT programme [44,45,46]. In a study among 395 subjects in an MMT programme, it was found that 6% used illicit drugs, which included stimulants, namely amphetamine, methamphetamine, and ecstasy, besides opioids [47]. The most reported reasons for use of illicit substances in individuals in the MMT programme are peer pressure, followed by drug craving [47]. Methamphetamine was found to be prevalent among MMT users, and most users report that they use it to increase energy and work performance [48,49]. It is therefore not unexpected that there is a higher likelihood of individuals in the MMT programme using illicit drugs to be also using ketum.

This study shows that the higher the Subjective Opiate Withdrawal Scale (SOWS), the more likely it is for an individual in the MMT programme to be a ketum user. Ketum is easily available, and considered cheaper among all illicit drugs [13]. Ketum, at low doses, has a stimulant effect, and at higher doses has an opioid-like effect [12]. There are many reasons why individuals use ketum in addition to other illicit substances. Some of them report using ketum concurrently with other illicit substances to get a “high” effect [50]. Other drug users use ketum as a substitute for an opioid to relieve withdrawal symptoms when they attempt to stop using the opioid, and as an alternative treatment for opioid dependence [13,15,42]. Therefore, it is possible that individuals with higher SOWS tend to use ketum as a self-remedy of withdrawal symptoms due to the sub-optimal methadone dose prescribed.

The Spearman ranked correlation test showed that there was a positive correlation between SOWS and KDS (rho = 0.570, *p* < 0.01) in this study. This shows that the more opioid withdrawal symptoms an individual exhibits, the more severe the ketum dependency. This study also found that there was also a negative mild correlation between methadone done and KDS. It is possible that the higher withdrawal symptoms could be related to sub-optimal methadone dose, subsequently leading to increased usage and dependence on ketum to substitute or curb the withdrawal symptoms. Hence, optimizing the methadone dose in an individual may reduce the opioid withdrawal symptoms, which leads to a reduction in both SOWS and KDS scores. However, the correlation between the methadone dose and KDS was not statistically significant (*p* > 0.05) as the sample size may probably not be big enough. Further studies are needed to investigate this hypothesis. Interestingly, this study showed that individuals with higher methadone doses (>60 mg daily) tend to use ketum in the MMT programme. However, this association was not statistically significant in the multivariate analysis. Some studies showed that prescribing a higher dose of methadone effectively reduced the opioids use in drug-dependent individuals, which these findings support the recommendations for a methadone maintenance therapy client to receive a daily methadone dose above 60 mg [51,52]. A possible explanation for why individuals tend to use ketum at higher methadone doses is that the individuals are using ketum to counteract the hypersedation adverse effect of higher doses of methadone. These individuals possibly use ketum for its stimulant effect. Methadone would block the euphoric effects of illicit opioid use once methadone reaches its optimal dose [53]. As the methadone fully occupies the brain opioids receptors, there is a diminished pleasurable effect of heroin use with higher methadone dose [53]. Hence, there is a possibility that individuals on higher doses of methadone are shifting from the use of heroin to another illicit substance with a stimulant effect, such as ketum, to get high or euphoric effects. Ketum has some affinity towards other receptors besides opioid receptors. Ketum may increase the euphoric effect by possibly acting on the dopamine receptors. However, further research is needed to prove the physiological significance.

There are a few limitations of this study. As this is a cross-sectional study, the authors could only determine if there was a presence of an association between the independent and dependent variables. The authors could not find the causative relationship between ketum use and its associating factors. Besides that, this study was conducted in a single setting. This might limit the generalizability of the study findings. The authors were using non-probable convenient sampling instead of random sampling in this study. Hence, this may limit the representative of the sample to the true population. A qualitative study would provide a much more flexible approach, allowing the authors to investigate more speculative reasons for subjects using ketum in the MMT programme.

## 5. Conclusions

In conclusion, this study demonstrated a high prevalence of ketum use in the MMT programme. To the knowledge of the authors, this was the first study that determined the prevalence of ketum use in individuals in the MMT programme and provided an evaluation of the association among the psychosocial factors affecting ketum use in individuals in the MMT programme. Ethnicity, duration in the MMT programme, other illicit drugs use, and SOWS were significantly associated with ketum use in the MMT programme in this study. Malay ethnicity is associated with a greater likelihood of becoming a ketum user compared to Chinese and Indian ethnicity in the MMT programme. The current study also showed that the longer an individual has been in the MMT programme is associated with a lesser risk of being a ketum user. This could be due to the effectiveness of the MMT programme in reducing the risk of individuals taking other illicit drugs (which includes ketum). Besides this, subjects who either exhibited higher SOWS or used other illicit drugs were more likely to use ketum. Further research would be required to explore causative factors for individuals in the MMT programme affecting ketum use.

## Figures and Tables

**Table 1:** Sociodemographic Data and Clinical Related Profiles of the Subjects (*n* = 215).

	Mean (SD)/Median (IQR)	*n* (%)
**Age**	45.0 (11.54) ^1^	
**Gender**		
Male		210 (97.7)
Female		5 (2.3)
**Ethnicity**		
Malay		142 (66.0)
Chinese		48 (22.3)
Indian		25 (11.6)
**Religion**		
Islam		144 (67.0)
Buddhist		46 (21.4)
Hinduism		19 (8.8)
Christianity		6 (2.8)
**Marital Status**		
Married		70 (32.6)
Widowed		3 (1.4)
Divorced/Separated		28 (13.0)
Never married		114 (53.0)
**Employment**		
Employed		164 (76.3)
Unemployed		51 (23.7)
**Accommodation**		
With family/friends		181 (84.2)
Stay alone		34 (15.8)
**Monthly household income (RM)**	1500.00 (1000.00) ^2^	
**Education**		
No formal education		2 (0.9)
Primary education		124 (57.7)
Secondary education		79 (36.7)
Tertiary education		10 (4.7)
**Duration in MMT programme (months)**	58 (90) ^2^	
**Current methadone dose (mg)**	65 (40) ^2^	
**Duration of heroin use before starting on MMT (years)**	18.5 (11.09) ^1^	
**Previous imprisonment**		
No		56 (26.0)
Yes		159 (74.0)
**Psychiatric illness**		
No		201 (93.5)
Yes		14 (6.5)
**Hepatitis B**		
No		213 (99.1)
Yes		2 (0.9)
**Hepatitis C**		
No		114 (53.0)
Yes		101 (47.0)
**HIV**		
No		209 (97.2)
Yes		6 (2.8)
**Other illicit drugs**		
No		18 (8.4)
Yes		197 (91.6)
**High-risk score (ASSIST)**		
No		206 (95.8)
Yes		9 (4.2)
**SOWS**	0 (1) ^2^	

^1^ = Mean (SD), ^2^ = Median (IQR); SD = Standard deviation; IQR = Interquartile range.

**Table 2:** Sociodemographic Data and Clinical Related Profiles Comparing Ketum Users and Non-Ketum Users.

	Ketum User			
	No (*n* = 109)		Yes (*n* = 106)	
	Mean (SD)/Median (IQR)	*n* (%)	Mean (SD)/Median (IQR)	*n* (%)
**Age**	50.3 (10.40) ^1^		39.5 (10.02) ^1^	
**Gender**				
Male		105 (96.3)		105 (99.1)
Female		4 (3.7)		1 (0.9)
**Ethnicity**				
Malay		48 (44.0)		94 (88.7)
Chinese		40 (36.7)		8 (7.5)
Indian		21 (19.3)		4 (3.8)
**Religion**				
Islam		47 (43.1)		97 (91.5)
Buddhist		41 (37.6)		5 (4.7)
Hinduism		16 (14.7)		3 (2.8)
Christianity		5 (4.6)		1 (0.9)
**Marital Status**				
Married		31 (28.4)		39 (36.8)
Widowed		2 (1.8)		1 (0.9)
Divorced/Separated		20 (18.3)		8 (7.5)
Never married		56 (51.4)		58 (54.7)
**Employment**				
Employed		79 (72.5)		85 (80.2)
Unemployed		30 (27.5)		21 (19.8)
**Accommodation**				
With family/friends		86 (78.9)		95 (89.6)
Stay alone		23 (21.1)		11 (10.4)
**Monthly household income (RM)**	1500.00 (1000.00) ^2^		1500.00 (1500.00) ^2^	
**Education**				
No formal education		1 (0.9)		1 (0.9)
Primary education		78 (71.6)		46 (43.4)
Secondary education		24 (22.0)		55 (51.9)
Tertiary education		6 (5.5)		4 (3.8)
**Duration in MMT programme (months)**	94.0 (84.0) ^2^		36.5 (76.0) ^2^	
**Current methadone dose (mg)**	60.0 (40.0) ^2^		70.0 (51.3) ^2^	
**Duration of heroin use before starting on MMT (years)**	21.5 (11.14) ^1^		15.4 (10.19) ^1^	
**Previous imprisonment**				
No		26 (23.9)		30 (28.3)
Yes		83 (76.1)		76 (71.7)
**Psychiatric illness**				
No		101 (92.7)		100 (94.3)
Yes		8 (7.3)		6 (5.7)
**Hepatitis B**				
No		107 (98.2)		106 (100.0)
Yes		2 (1.8)		0 (0.0)
**Hepatitis C**				
No		46 (42.2)		68 (64.2)
Yes		63 (57.8)		38 (35.8)
**HIV**				
No		104 (95.4)		105 (99.1)
Yes		5 (4.6)		1 (0.9)
**Other illicit drugs**				
No		17 (15.6)		1 (0.9)
Yes		92 (84.4)		105 (99.1)
**High-risk score (ASSIST)**				
No		108 (99.1)		98 (92.5)
Yes		1 (0.9)		8 (7.5)
**SOWS**	0 (0) ^2^		0 (3) ^2^	

^1^ = Mean (SD), ^2^ = Median (IQR); SD = Standard deviation; IQR = Interquartile range.

**Table 3:** Spearman Ranked Correlation between SOWS, Methadone Dose and KDS (*n* = 215).

	SOWS	Methadone Dose (mg)	KDS (*n* = 106)
**SOWS**	-	0.069	0.570 **
**Methadone Dose (mg)**	0.069	-	−0.106
**KDS (n = 106)**	0.570 **	−0.106	-

** *p* value < 0.01.

**Table 4:** Univariate Analysis of Factors Associated with Ketum Use.

	Ketum User		Statistical Test	Odds Ratio (95% CI)	*p* Value
	No (*n* = 109)	Yes (*n* = 106)			
**Age, Mean (SD)**	50.3 (10.40)	39.5 (10.02)	7.753 ^t^	-	<0.01
**Gender, n (%)**					
Male (R)	105 (96.3)	105 (99.1)			
Female	4 (3.7)	1 (0.9)	1.758 ^a^	0.250 (0.027, 2.274)	0.369
**Ethnicity, n (%)**					
Malay (R)	48 (44.0)	94 (88.7)			
Chinese	40 (36.7)	8 (7.5)	28.684 ^b^	0.102 (0.044, 0.235)	<0.01
Indian	21 (19.3)	4 (3.8)	16.501 ^b^	0.097 (0.032, 0.299)	<0.01
**Religion, n (%)**					
Islam (R)	47 (43.1)	97 (91.5)			
Buddhist	41 (37.6)	5 (4.7)	31.259 ^b^	0.059 (0.022, 0.159)	<0.01
Hinduism	16 (14.7)	3 (2.8)	13.460 ^b^	0.091 (0.025, 0.327)	<0.01
Christianity	5 (4.6)	1 (0.9)	4.423 ^b^	0.097 (0.011, 0.853)	0.035
**Marital Status, n (%)**					
Married (R)	31 (28.4)	39 (36.8)			
Widowed/Divorced/Separated/Never Married	78 (71.6)	67 (63.2)	1.707 ^c^	0.683 (0.385, 1.212)	0.191
**Employment, n (%)**					
Employed (R)	79 (72.5)	85 (80.2)			
Unemployed	30 (27.5)	21 (19.8)	1.766 ^c^	0.651 (0.344, 1.229)	0.184
**Accommodation, n (%)**					
With family/friends (R)	86 (78.9)	95 (89.6)			
Stay alone	23 (21.1)	11 (10.4)	4.642 ^c^	0.433 (0.199, 0.940)	0.031
**Monthly household income (RM), Median (IQR)**	1500.00 (1000.00)	1500.00 (1500.00)	−0.627 ^u^	-	0.531
**Education, n (%)**					
≤Primary education (R)	79 (72.5)	47 (44.3)			
>Primary education	30 (27.5)	59 (55.7)	17.538 ^c^	3.306 (1.872, 5.838)	<0.01
**Duration in MMT Programme (months), Median (IQR)**	94.0 (84.0)	36.5 (76.0)	−4.724 ^u^	-	<0.01
**Methadone dose (mg)**					
≤60 (R)	60 (55)	42 (39.6)			
>60	49 (45)	64 (60.4)	5.127 ^c^	1.866 (1.085, 3.209)	0.024
**Duration of heroin use before starting on MMT (years), Mean (SD)**	21.5 (11.14)	15.4 (10.19)	4.184 ^t^	-	<0.01
**Previous Imprisonment**, **n (%)**					
No (R)	26 (23.9)	30 (28.3)			
Yes	83 (76.1)	76 (71.7)	0.552 ^c^	0.794 (0.431, 1.461)	0.457
**Psychiatric illness, n (%)**					
No (R)	101 (92.7)	100 (94.3)			
Yes	8 (7.3)	6 (5.7)	0.249 ^c^	0.758 (0.254, 2.262)	0.618
**Hepatitis B, n (%)**					
No (R)	107 (98.2)	106 (100.0)			
Yes	2 (1.8)	0 (0.0)	1.963 ^a^	0.502 (0.439, 0.574)	0.498
**Hepatitis C, n (%)**					
No (R)	46 (42.2)	68 (64.2)			
Yes	63 (57.8)	38 (35.8)	10.394 ^c^	0.408 (0.236, 0.707)	0.001
**HIV, n (%)**					
No (R)	104 (95.4)	105 (99.1)			
Yes	5 (4.6)	1 (0.9)	2.630 ^a^	0.198 (0.023, 1.725)	0.212
**Other illicit drugs, n (%)**					
No (R)	17 (15.6)	1 (0.9)			
Yes	92 (84.4)	105 (99.1)	15.041 ^c^	19.402 (2.533, 148.636)	<0.01
**High-risk score (ASSIST)**					
No (R)	108 (99.1)	98 (92.5)		8.816 (1.083, 71.763)	
Yes	1 (0.9)	8 (7.5)	5.889 ^a^		0.018
**SOWS**	0 (0)	0 (3)	−5.356 ^u^	-	<0.01

CI = Confidence interval; (R) = Reference group; ^t^ = *t* test; ^a^ = Fisher’s Exact test; ^b^ = Wald Chi Square; ^c^ = Pearson Chi Square; ^u^ = Mann-Whitney U test.

**Table 5:** Multivariate Analysis of Factors Associated with Ketum Use.

	Adjusted Odds Ratio (95% CI)	*p* Value
**Ethnicity**		0.002
Chinese	0.386 (0.134, 1.113)	0.078
Indian	0.119 (0.035, 0.408)	0.001
Malay (R)		
**Accommodation**		
Stay alone	0.610 (0.219, 1.699)	0.344
With family/friends (R)		
**Education**		
>Primary education	1.958 (0.940, 4.081)	0.073
≤Primary education (R)		
**Duration in MMT Programme (months)**	0.990 (0.982, 0.998)	0.012
**Methadone dose (mg)**		
>60	1.153 (0.387, 3.432)	0.798
≤60 (R)		
**Duration of heroin use before starting on MMT (years)**	0.974 (0.939, 1.009)	0.147
**Hepatitis C**		
Yes	0.806 (0.377, 1.724)	0.578
No (R)		
**Other illicit drugs**		
Yes	9.914 (1.109, 88.602)	0.040
No (R)		
**High-risk score (ASSIST)**		
Yes	1.607 (0.151, 17.045)	0.694
No (R)		
**SOWS**	1.340 (1.070, 1.677)	0.011

CI = Confidence interval; (R)—Reference group.

**Table 6:** Descriptive Statistics of Other Illicit Drugs.

	Ketum User			
	No (*n* = 109)		Yes (*n* = 106)	
	n	%	n	%
**Cannabis**				
No	45	41.3	19	17.9
Yes	64	58.7	87	82.1
**Stimulant**				
No	42	38.5	9	8.5
Yes	67	61.5	97	91.5
**Inhalant**				
No	106	97.2	83	78.3
Yes	3	2.8	23	21.7
**Sedative/hypnotic**				
No	67	61.5	61	57.5
Yes	42	38.5	45	42.5
**Hallucinogen**				
No	101	92.7	88	83
Yes	8	7.3	18	17

## Data Availability

The data presented in this study are available on request from the corresponding author. The data are not publicly available due to privacy and ethical issues.
